# Effect of baseplate size on primary glenoid stability and impingement-free range of motion in reverse shoulder arthroplasty

**DOI:** 10.1186/1471-2474-15-417

**Published:** 2014-12-09

**Authors:** Soo-Won Chae, Soung-Yon Kim, Haea Lee, Joung-Ro Yon, Juneyoung Lee, Seung-Ho Han

**Affiliations:** Department of Mechanical Engineering, Korea University, Seoul, South Korea; Department of Orthopaedic Surgery, Hallym University, Kangnam Sacred Heart Hospital, Seoul, South Korea; Department of Biostatistics, Korea University College of Medicine, Seoul, South Korea; Department of Anatomy, College of Medicine, Chung-Ang University, Seoul, South Korea

**Keywords:** Reverse shoulder arthroplasty, Smaller baseplate, Biomechanical testing, Simulated computer model

## Abstract

**Background:**

Use of a baseplate with a smaller diameter in reverse shoulder arthroplasty is increasing, especially in patients with a small glenoid or glenoid wear. However, the effect of a smaller baseplate on stability of the glenoid component has not been evaluated. Thus, the purpose of this study was to determine whether a smaller baseplate (25 mm) is beneficial to the initial stability of the glenoid component compared to that with a baseplate of a commonly used size (29 mm).

**Methods:**

Micromotion of glenoid components attached to 14 scapulae of fresh-frozen cadavers was measured and compared between 25- and 29-mm baseplates in biomechanical testing. Impingement-free range of motion in abduction, adduction, internal rotation, and external rotation was evaluated by using a simulated computer model constructed based on the same fresh-frozen cadavers used in biomechanical testing.

**Results:**

Micromotion at the inferior third of the glenoid-glenosphere interface was higher in the 29-mm baseplate group than in the 25-mm baseplate group during both 0.7- and 1-body weight cyclic loading in biomechanical testing. Adduction deficit was smaller, and total impingement-free range of motion from abduction to adduction and rotation were greater in the 25-mm baseplate group than in the 29-mm baseplate group in the simulated computer model.

**Conclusions:**

Use of a baseplate with a smaller diameter (25 mm) in reverse shoulder arthroplasty is suitable for improving the primary stability of the glenoid component. With a smaller baseplate, impingement-free range of motion is optimized in a smaller glenoid.

**Electronic supplementary material:**

The online version of this article (doi:10.1186/1471-2474-15-417) contains supplementary material, which is available to authorized users.

## Background

Since its introduction by Grammont [1, 2], reverse shoulder arthroplasty (RSA) has been successful in improving function and pain in patients with massive rotator cuff tear or cuff tear arthropathy [2–4]. Recently, its application has expanded to include rheumatoid arthritis, fracture, failed arthroplasty, and fracture and infection sequelae [5–7]. However, the reported complication rate is still high [8]. Glenoid component loosening, in particular, is known to be a major cause of failure in RSA [9]. There have been several efforts to improve stability of glenoid component fixation, including studies of glenoid component design [10, 11], glenoid component position [12], and screw fixation [13]. During the RSA procedure, because of glenoid wear or small glenoid size, glenoid surface area, which is required for seating of the baseplate, is sometimes insufficient to fix the commonly used 29-mm-diameter baseplate [14, 15]. Surgeons doubt the stability of initial fixation of the baseplate when confronted with this difficulty. Facing this uncertainty, some manufacturers have developed baseplates with a smaller diameter, and their use is increasing, especially among small, female, and Asian patients. However, the effect of a smaller baseplate, the stability of a smaller baseplate compared to that of a baseplate with a commonly used size, has not been examined biomechanically. Thus, the purpose of this study was to determine whether a 25-mm baseplate is beneficial to initial stability of the glenoid component and impingement-free range of motion in a relatively small glenoid, compared to that with a 29-mm baseplate. We hypothesized that the smaller 25-mm baseplate would provide better initial fixation stability, lesser micromotion, and more impingement-free range of motion than the commonly used 29-mm baseplate. This was evaluated by comparing micromotion at the glenoid-glenosphere interface in biomechanical testing and impingement-free range of motion in abduction, adduction, internal rotation, and external rotation in a simulated computer model of fresh-frozen cadavers with a small glenoid.

## Methods

### Biomechanical testing

Seven pairs of human scapulae were dissected from 7 fresh-frozen female cadavers with a mean age of 62 years (range, 61 to 65 years) and height less than 160 cm. We selected female donors with a small height because a small glenoid was preferable for our experimental purpose [16]. All cadavers investigated in this study were donated to The Catholic University of Korea by due process of law in Korea with the permission of the individuals prior to death and their family for dissection in educational and research settings. The IRB of The Catholic University of Korea has received AAHRPP accreditation and complies with its ethics. The IRB does not review cadaver studies in which it is not possible to identify the individuals and which do not use personal information. Research involving cadavers, autopsy material or bio-specimens from deceased individuals do not meet the regulatory definitions of human research and do not need IRB review. Our cadaver study complied with these conditions. Specimens showing posttraumatic deformity or degenerative change, such as glenoid wear, based on gross inspection, were excluded. Maximum length of the glenoid from the highest to the lowest point and maximum width of the anteroposterior diameter of the glenoid were measured using a caliper.

A Tornier Aequalis Reversed Shoulder Prosthesis (Tornier, Inc., Edina, MN, USA), consisting of a baseplate and glenosphere, was fixed to each scapula according to the standard surgical technique. Specimens were randomly assigned to 2 groups. The control group consisted of 7 scapulae implanted with a 29-mm baseplate and 36-mm glenosphere. The second group consisted of 7 scapulae implanted with a 25-mm baseplate and 36-mm glenosphere. The glenoid was reamed at neutral inclination. The version of the glenoid component was 0°, and the baseplate was positioned as inferiorly as possible relative to the scapula while still being fully supported by bone. In the same manner as in clinical surgery, the implantation of the baseplate and glenosphere at neutral inclination was accomplished using the neutral tilt central guide hole included with the prosthesis instrumentation. This guide hole allowed the insertion of a guide pin tilted 0° neutral relative to the glenoid surface. To align the lower border of the baseplate with the inferior glenoid rim, the drill holes were positioned at 11.5 and 9.5 mm above the inferior glenoid rim for the 29- and 25-mm baseplates prior to the glenoid reaming respectively, in compliance with the 12-mm rule described by Kelly *et al*. [17].

Screw fixation was performed according to the manufacturer’s recommended surgical technique. The anterior screw was inserted at a trajectory that was superior and towards the middle of the baseplate. The posterior screw was inserted at a trajectory that was inferior and towards the middle of the baseplate. The inferior screw was positioned into the pillar of the scapula and was generally situated downwards in the vertical axis of the glenoid at an angle of ~20°. The superior screw was positioned into the base of the coracoid process and was generally situated superiorly in the vertical axis of the glenoid at an angle of ~20° and anteriorly in the transverse axis of the glenoid at an angle of ~10°. We attempted to fix the 25- and 29-mm baseplates in the same directions as the screws.

**Figure 1:**
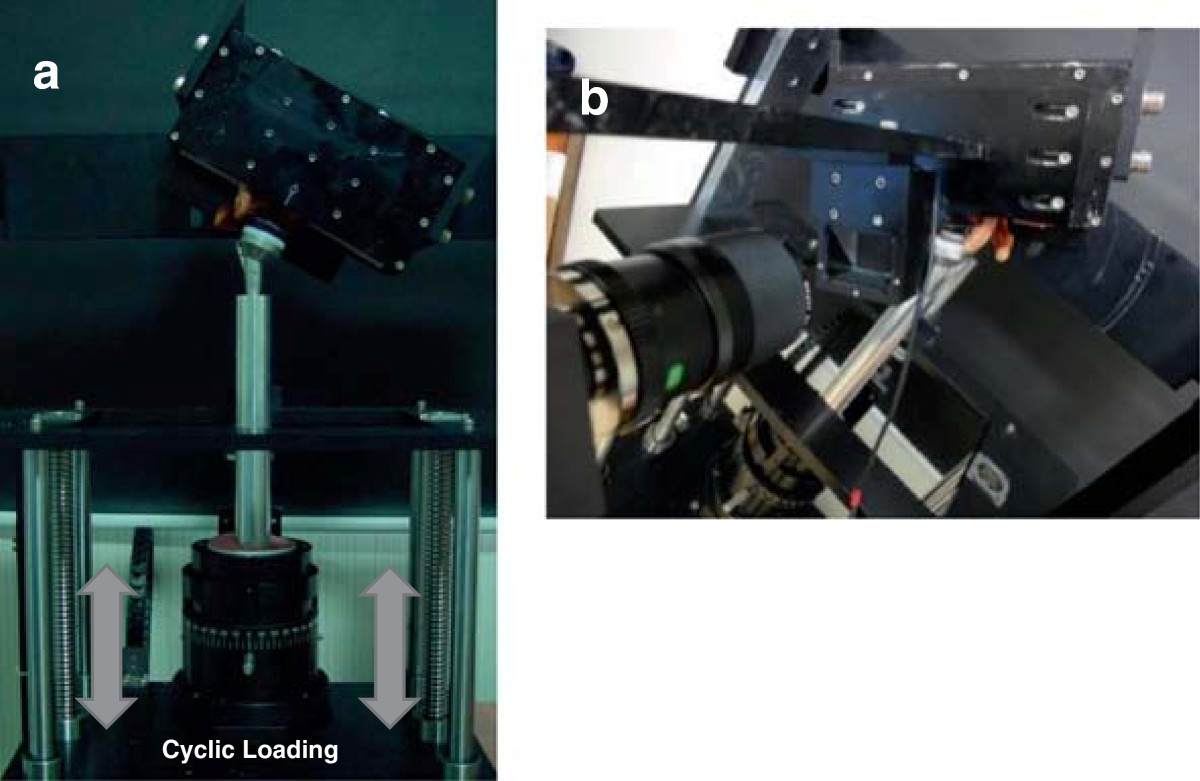
**Actual experimental setup. (a)** The specimen was mounted on a custom-made axial-compressive loading machine. **(b)** The camera was connected by a frame and positioned perpendicular to the markers where micromotion was measured.

**Figure 2:**
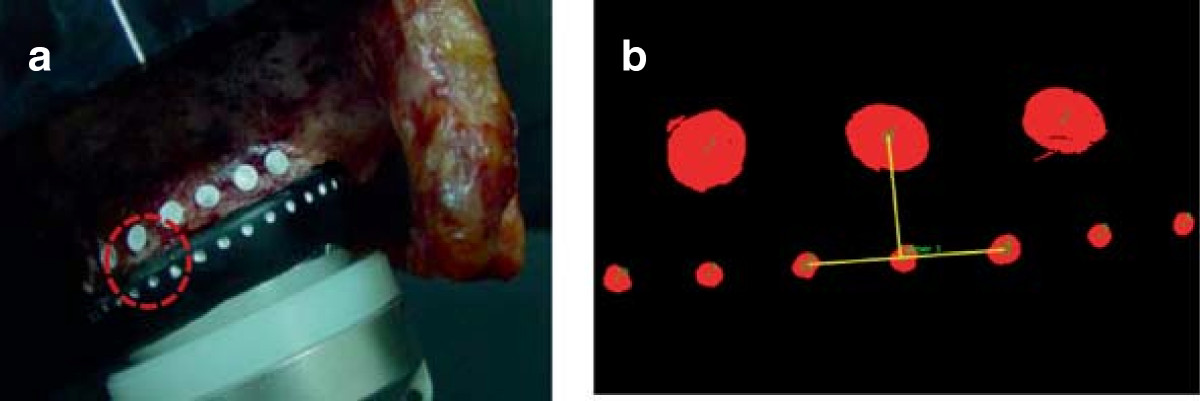
**Micromotion measurement. (a)** Four surface markers were placed at the inferior third of the glenoid-glenosphere interface for measuring micromotion. **(b)** Micromotion was assessed using high-resolution digital imaging.

The scapula with the implant was embedded in a rectangular resin block (Lang Dental Manufacturing Co., Inc., Wheeling, IL, USA) so that the glenoid-glenosphere interface was parallel to the floor. The block was then bolted to the mounting plate on a custom-made axial-compressive loading machine. The humeral component of the prosthesis, consisting of a 6-mm polyethylene insert and 36-mm metaphysis and stem, was provisionally affixed to the machine. The specimen was mounted on the axial-compressive loading machine at 60° abduction of the glenoid component to the humeral component (Figure 1a), and 2 compressive cyclic loading modes were applied sequentially through the humeral cup assembly. Cyclic loads of 0.7 body weight (BW) (480 N, 2.5 Hz, 100 cycles) and then that of 1 BW (686 N, 2.5 Hz, 100 cycles) were applied parallel with the long axis of the humeral stem to the center of the glenosphere. We assessed micromotion at the inferior third of the glenoid-glenosphere interface using high-resolution digital imaging [18]. For micromotion analysis, laser markers (1-mm diameter) were engraved on the glenosphere surface prior to implantation such that there was a 3-mm distance between each marker and a 1-mm distance from the rim of the glenosphere. Surface markers (polyvinyl chloride, 2-mm diameter) were affixed with super glue to the adjacent glenoid bone surface near the glenoid-glenosphere interface. A camera was connected by a frame to the resin box in which the scapula with the implant was embedded, and was positioned perpendicular to the markers where micromotion was measured (Figure 1b). Four surface markers placed at the inferior third of the glenoid-glenosphere interface (one on the surface of the glenoid bone and 3 on the surface of the glenosphere) were used for measuring micromotion during cyclic loading in biomechanical testing (Figure 2a). Micromotion of the glenoid component was defined as the difference in glenoid component displacement from the adjacent glenoid bone surface. Locations of the markers were recorded using a camera (Pearl CCD series; IMI-Technology, San Diego, CA, USA) and 2/3-inch 55-mm telecentric lens (TEC-M55; Computar, NY, USA), and micromotion data were collected using a custom-made LabVIEW graphic interface (National Instruments Corporation, Austin, TX, USA). Micromotion, both parallel (x-axis) and perpendicular (y-axis) to the glenoid-glenosphere interface, was assessed as the hypotenuse of x- and y-axes measured during biomechanical testing (Figure 2b).

### Simulated computer model

Scapulae from the same fresh-frozen cadavers used in biomechanical testing were used for computer model simulation. Computed tomography scans of each scapula were obtained, and 1-mm slices (resolution, 0.488-mm pixel size) were used to construct 3-dimensional (3D) models using Mimics software (Materialise, Leuven, Belgium). A corresponding 3D prosthesis model consisting of the baseplate (25- or 29-mm) and 36-mm glenosphere was generated by laser scanning (Rexcan 3D Laser Scanner; Solutionix, Seoul, Korea). Point data of the scapula from Mimics and of the implant from the 3D laser scanner were converted into surface data using a reverse engineering program (Rapidform 3D Systems, Inc., Rock Hill, SC, USA). The scapular plane was defined as connecting the most dorsal aspect of the inferior angle, the intersection of the scapular spine and medial border, and the center of the glenoid (intersection of vertical and horizontal glenoid midlines) [14, 19]. We computed implantation of the glenoid component into the scapula as the same condition in each group in biomechanical testing.

Humeral abduction, adduction, internal rotation, and external rotation in relation to the glenoid in the scapular plane were simulated using SolidWorks 2011 software (SolidWorks Corporation, Concord, MA, USA). Impingement-free abduction and adduction arc of motion were defined as the arc of motion from the point of inferior impingement to the point of superior impingement on the scapula or acromion [20]. Impingement-free range of motion in internal and external rotation was measured at 60° abduction of the humeral component to the glenoid component.

### Data analysis

Differences in micromotion of the glenoid component during cyclic loading in biomechanical testing and impingement-free range of motion in the simulated computer model between groups were analyzed using Wilcoxon’s rank-sum test. Data were presented as means ± SDs. Reported *p* values were 2-tailed, and values <0.05 were considered to be statistically significant.

## Results

**Figure 3:**
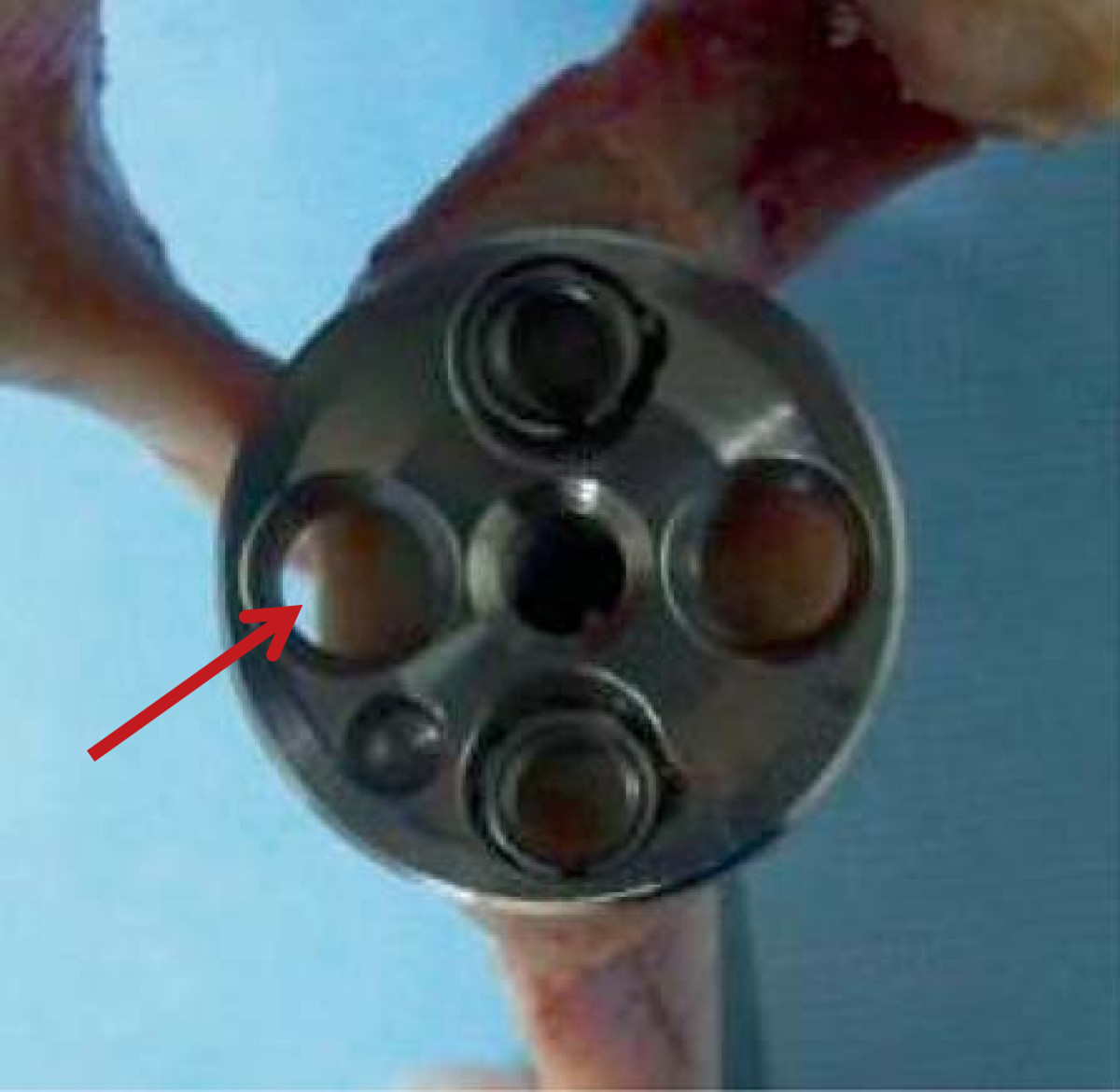
**A 29-mm baseplate.** This commonly used baseplate (29 mm) is too large to fix on a small glenoid. The arrow indicates insufficient bone stock for screw fixation.

Gross inspection showed that all glenoids had a smaller width than the 29-mm baseplate diameter, as well as insufficient bone stock to fix the anterior or posterior screw for fixation of the 29-mm baseplate (Figure 3).

Measurements of glenoid size and screw length are listed in Table 1. The lengths of the anterior, posterior, and superior screws were longer in the 25-mm baseplate group than in the 29-mm baseplate group. Micromotion at the inferior third of the glenoid-glenosphere interface was higher in the 29-mm baseplate group than in the 25-mm baseplate group during both 0.7-BW (34 ± 12 μm and 30 ± 6 μm, respectively *p* = 0.73) and 1-BW (47 ± 18 μm and 43 ± 10 μm, respectively; *p* = 0.91) cyclic loading. However, no statistically significant difference was evident between groups during the 0.7- or 1-BW cyclic loading.

**Table 1:** **Measurements of glenoid size and length of screws used for baseplate fixation**

Variable, mm	25-mm baseplate	29-mm baseplate	***p*** value
Glenoid length	32.6 ± 2.5	32.1 ± 2.4	0.64
Glenoid width	23.3 ± 2.0	23.3 ± 1.7	0.96
Anterior screw	22.3 ± 4.9	19.4 ± 4.7	0.34
Posterior screw	20.6 ± 7.8	16 ± 0	0.46
Superior screw	32 ± 6.4	25.4 ± 5.8	0.09
Inferior screw	31.1 ± 7.4	32.6 ± 6.7	0.78

All data are expressed as mean ± SD.

**Figure 4:**
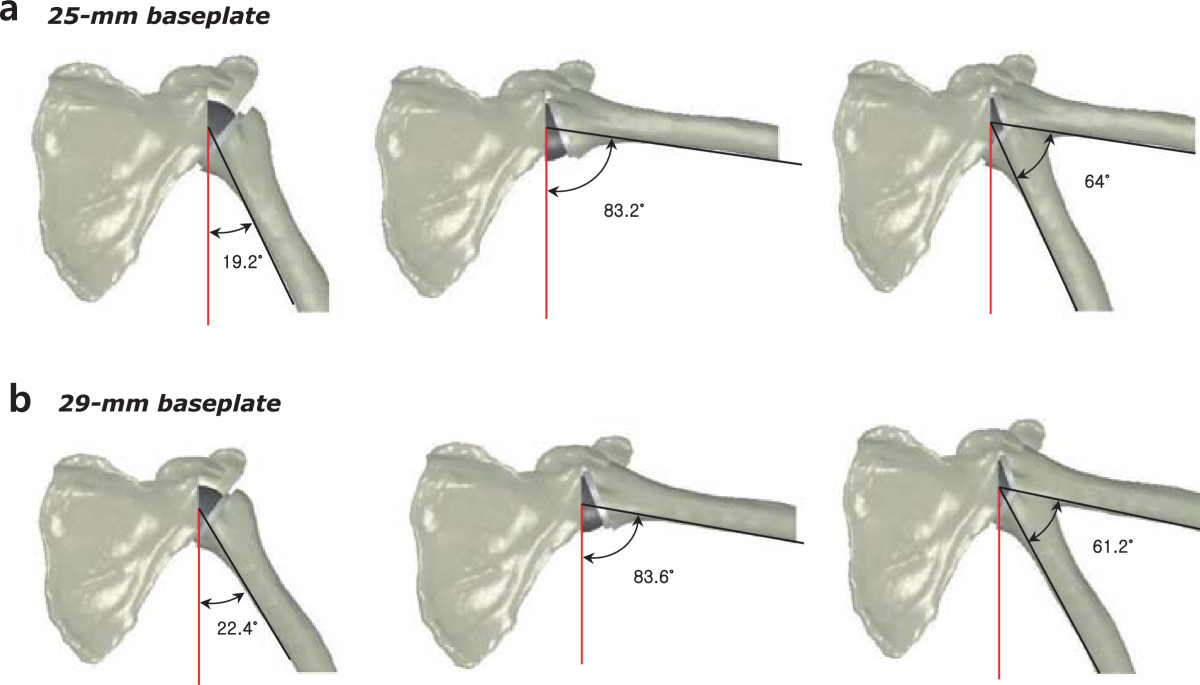
**Simulated computer model based on the same fresh-frozen cadavers used in biomechanical testing. (a)** Adduction deficit, maximum impingement-free abduction, and total impingement-free arc of motion in the 25-mm baseplate group. **(b)** Adduction deficit, maximum impingement-free abduction, and total impingement-free arc of motion in the 29-mm baseplate group.

**Figure 5:**
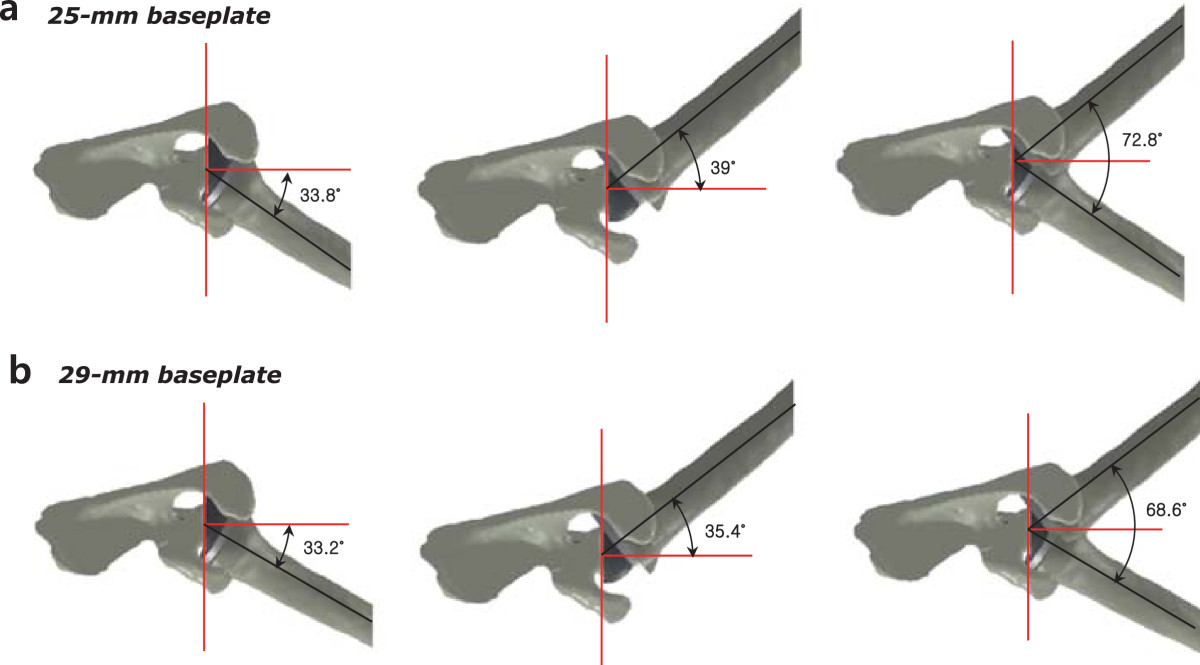
**Simulated computer model based on the same fresh-frozen cadavers used in biomechanical testing. (a)** Impingement-free range of motion in internal rotation, external rotation, and total impingement-free arc of rotation at 60° abduction in the 25-mm baseplate group. **(b)** Impingement-free range of motion in internal rotation, external rotation, and total impingement-free arc of rotation at 60° abduction in the 29-mm baseplate group.

Adduction deficit was smaller in the 25-mm baseplate group than in the 29-mm baseplate group (19.2° ± 4.9° and 22.4° ± 5.7°, respectively; *p* = 0.83), whereas total impingement-free range of motion from abduction to adduction was greater in the 25-mm baseplate group than in the 29-mm baseplate group (64° ± 3.8° and 61.2° ± 3.9°, respectively; *p* = 0.24). Maximum impingement-free abduction was similar between the 25- and 29-mm baseplate groups (83.2° ± 1.9° and 83.6° ± 2.1°, respectively; *p* = 0.48) (Figure 4). Impingement-free range of motion in external rotation was greater in the 25-mm baseplate group than in the 29-mm baseplate group (39° ± 8.1° and 35.4° ± 7.7°, respectively; *p* = 0.20), whereas impingement-free range of motion in internal rotation was similar between the 25- and 29-mm baseplate groups (33.8° ± 5.8° and 33.2° ± 7.0°, respectively; *p* = 1.00). Total impingement-free range of motion in rotation was greater in the 25-mm baseplate group than in the 29-mm baseplate group (72.8° ± 5.3° and 68.6° ± 2.8°, respectively; *p* = 0.31) (Figure 5). A statistically significant difference was not observed for these various parameters.

## Discussion

Numerous studies have suggested several methods to increase glenoid component stability and reduce scapular notching, such as glenoid component design [10, 11, 20–23], positioning [12, 24, 25], and screw fixation [13, 26, 27]. Besides glenoid implant factors, another consideration to improve glenoid component fixation is glenoid morphology and size. The glenoid morphology of patients undergoing RSA is heterogeneic in terms of bony deformity, including posterior, superior, global, and anterior wear [14]. Glenoid wear may result in decreased contact surface area and bony support to the baseplate, which may weaken initial stability of glenoid component fixation.

Several literatures have reported anatomic measurements of the glenoid. Churchill et al. reported that male glenoid width and height were 27.8 ± 1.6 mm and 37.5 ± 2.2 mm, respectively, while female glenoid width and height were 23.6 ± 1.5 mm and 32.6 ± 1.8 mm, respectively. Differences between men and women were found to be significant [16]. Von Schroeder et al. reported that average glenoid dimensions were 29 ± 3 mm (anteroposterior) by 36 ± 4 mm (superoinferior) [28]. Glenoid size, especially in Asian women, is sometimes smaller than the commonly used 29-mm baseplate; therefore, the need for appropriate sizing of the glenoid component has been suggested. Kim et al. reported that in the Korean population, mean glenoid radius of women over 60 years of age was 13.5 ± 1.7 mm; thus it is difficult to insert a 29-mm baseplate (radius, 14.5 mm) in this small glenoid [15]. In small glenoids, the standard glenoid implant (29-mm) is larger than glenoid bone stock, which results in insufficient bone-implant contact and screw fixation, especially of anterior and posterior holes of the baseplate. Initial rigid fixation of the glenoid baseplate is dependent on placement of the screws and glenoid bone stock [10]; insecure initial fixation due to inadequate bony support to the baseplate and screw fixation is likely to result in glenoid loosening and decreased longevity of the RSA.

In theory, a smaller diameter baseplate would serve to improve initial glenoid component fixation in terms of contact surface area and bony support to the baseplate. Clinically, its use has been increasing, especially in small, female, and Asian patients. Middernacht et al. also suggested use of a baseplate with a smaller radius than currently used to avoid scapular notching, considering the varied size of the infraglenoid tubercle. They recommended using a smaller baseplate than is currently available in order to move the center of rotation even lower on the glenoid face [29, 30]. Despite several authors suggesting use of a smaller baseplate to reduce scapular notching or to adjust a small glenoid [15, 29, 30], and its use increasing in smaller patients, the effect of a smaller baseplate with respect to initial fixation strength has not been thoroughly investigated.

The present study aimed to clarify whether use of a smaller baseplate (25 mm) is beneficial to improve initial stability of glenoid component fixation in a small glenoid, compared with the commonly used baseplate (29 mm). Our results demonstrated that the smaller baseplate had less micromotion and greater impingement-free range of motion, even though a statistically significant difference was not evident. We postulate that because fixation of a 25-mm baseplate with a 36-mm glenosphere creates an overhang of 5.5 mm, while fixation of a 29-mm baseplate with a 36-mm glenosphere creates an overhang of 3.5 mm, this specific design of the prosthesis may affect adduction deficit as well as total impingement-free range of motion. Based upon our observations, we assumed the shorter length of the anterior, posterior, and superior screws used for the 29-mm baseplate fixation (relative to those used for the 25-mm baseplate fixation) and the insufficient bone stock of the small glenoid for fixation of the larger 29-mm baseplates, particularly for the fixation of the anterior and posterior screws, might influence the biomechanical stability of the 29-mm baseplate on the small glenoid.

Some strengths of this study include the use of fresh-frozen cadavers reflecting small glenoid morphology and density of cortical and cancellous scapula bone in individuals aged over 60 years. Use of fresh-frozen cadavers with a small glenoid, representing the population in which cuff tear arthropathy commonly occurs, better reflects the anatomic and mechanical properties of glenoid bone, enabling better assessment of glenoid component stability in RSA. In addition, we used digital-image analysis of micromotion in biomechanical testing, which is more reliable than the gauge method [18]. Furthermore, we evaluated the influence of baseplate size in RSA multiply by assessing micromotion in biomechanical testing and impingement-free abduction and adduction arc of motion in a simulated computer model. To our knowledge, no study has assessed stability and impingement-free arc of motion related to micromotion and scapular notching overall.

There are some limitations associated with the experimental setup and methodology. First, the 0.7- and 1-BW amounts of loading used in this study could be deemed high considering the forces or load values estimated to occur in the shoulder during normal daily activities [31–33]. In addition, we applied relatively few loading cycles to simulate shoulder mechanics. Considering the high amount of axial-compressive loading forces used in our study, we believe the shorter-duration test might not have influenced the results concerning initial fixation stability of the glenoid component [11, 18]. Second, we did not use cadaveric shoulders with rotator cuff arthropathy, which is the most common indication for RSA. In rotator cuff arthropathy, glenohumeral articulation is altered and glenoid wear commonly develops. Third, we assessed glenoid component stability only at an angle of 60° abduction. Different angle of loading condition may affect stability of the glenoid component. Fourth, we evaluated the influence of a smaller baseplate by using one specific type of prosthesis. Different prosthesis designs, such as shape of the baseplate, eccentricity, and curvature of the glenosphere, as well as number or size of screws for fixation, may affect primary stability and/or impingement-free arc of motion in RSA. Finally, stabilizing or destabilizing effects of ligaments, joint capsule, or remaining rotator cuff muscles, which may affect biomechanics in RSA, were not considered.

## Conclusions

This study demonstrated that a smaller baseplate (25 mm) could be suitable for improving the primary stability of the glenoid component as well as impingement-free range of motion in a relatively small glenoid, compared with the commonly used baseplate (29 mm). Use of a smaller baseplate could be optimal in a small glenoid and may make fixation of the glenoid component more stable and may result in more impingement-free range of motion. We believe that further clinical and basic research studies should be performed to assess the influence of baseplate size on the stability of fixation in RSA, considering various designs of the glenoid component.

## Authors’ original submitted files for images

Below are the links to the authors’ original submitted files for images. Authors’ original file for figure 1 Authors’ original file for figure 2 Authors’ original file for figure 3 Authors’ original file for figure 4 Authors’ original file for figure 5

## Abbreviations

RSA: Reverse shoulder arthroplasty.d
