# Supplementary figures and images for: Effect of baseplate size on primary glenoid stability and impingement-free range of motion in reverse shoulder arthroplasty
Source: BMC Musculoskelet Disord. 2014 Dec 9;15:417. doi: 10.1186/1471-2474-15-417 (PMC4295354; doi:10.1186/1471-2474-15-417)

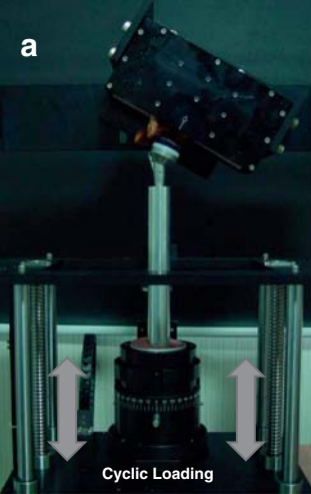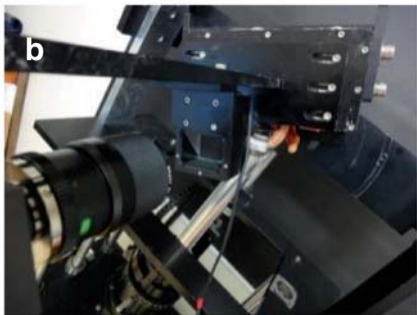

Supplement: Supplementary file 1 — Authors’ original file for figure 1 [file 12891_2014_2350_MOESM1_ESM.pdf]

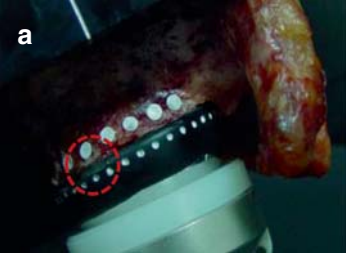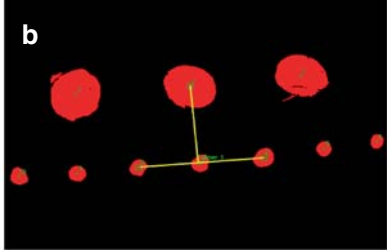

Supplement: Supplementary file 2 — Authors’ original file for figure 2 [file 12891_2014_2350_MOESM2_ESM.pdf]

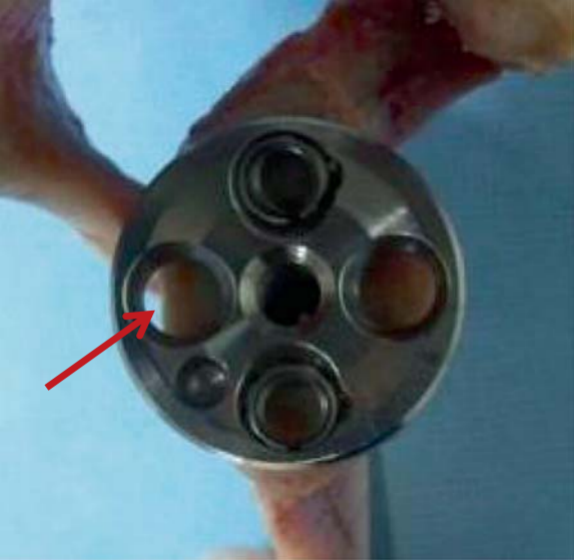

Supplement: Supplementary file 3 — Authors’ original file for figure 3 [file 12891_2014_2350_MOESM3_ESM.pdf]

**a** 25-mm baseplate

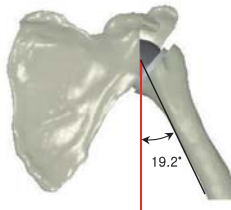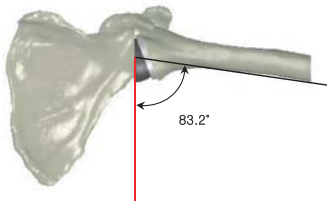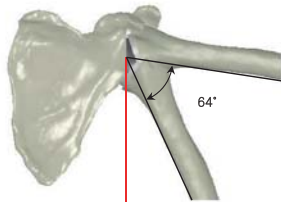

**b** 29-mm baseplate

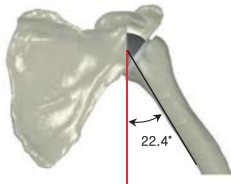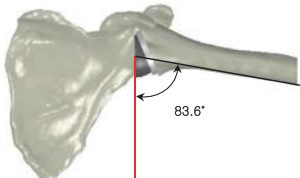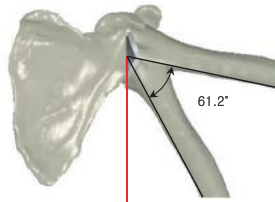

Supplement: Supplementary file 4 — Authors’ original file for figure 4 [file 12891_2014_2350_MOESM4_ESM.pdf]

**a** 25-mm baseplate

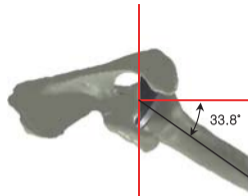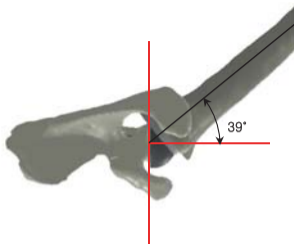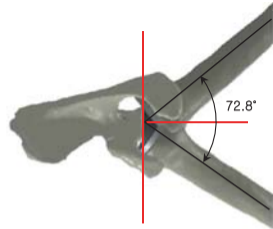

**b** 29-mm baseplate

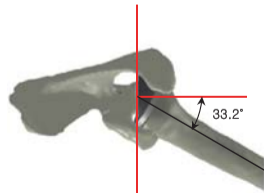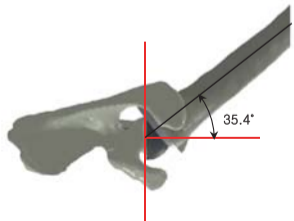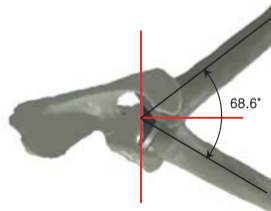

Supplement: Supplementary file 5 — Authors’ original file for figure 5 [file 12891_2014_2350_MOESM5_ESM.pdf]
